# Impact of supportive bra use and surgical factors on seroma and pain following breast-conserving surgery: a single-center retrospective study

**DOI:** 10.25122/jml-2026-0035

**Published:** 2026-06

**Authors:** Sümeyra Emine Bölük, Salih Bölük, Çağrı Bilgiç, Özlem Akoğlu

**Affiliations:** 1 Sultan Abdulhamid Han Training and Research Hospital, Republic of Turkey Ministry of Health, İstanbul, Türkiye; 2 Gebze Medical Park Hospital, Gebze, Türkiye

**Keywords:** breast-conserving surgery, seroma formation, postoperative pain, supportive bra

## Abstract

Breast-conserving surgery (BCS) is widely accepted as a standard surgical approach in early-stage breast cancer. However, postoperative complications such as pain and seroma formation remain frequent and may negatively affect quality of life. The potential benefits of supportive bra use in the postoperative period have been suggested but remain insufficiently investigated. This single-center retrospective study included 128 female patients who underwent upfront BCS. All patients were advised to wear a supportive bra for at least 16 hours daily for 3 months postoperatively. Patients were categorized into two groups: regular supportive bra users (*n* = 74) and non-users (*n* = 54). Pain was assessed using the Visual Analog Scale (VAS) at postoperative week 1, month 1, month 6, and year 1. Seroma was evaluated using clinical and ultrasonographic assessments at 1 year. Statistical analyses were performed using nonparametric tests. Supportive bra users demonstrated significantly lower VAS scores at week 1, month 1, and month 6 (*P* = 0.001 for all comparisons). No significant difference was observed at year 1 (*P* = 0.052). At 1 year, the absence of seroma was significantly higher among supportive bra users (32.4%) than among non-users (18.5%) (*P* = 0.025). Regular use of a supportive bra after BCS was associated with significantly reduced postoperative pain and improved long-term seroma resolution. Given its non-invasive nature and low cost, supportive bra use may represent a simple and effective strategy to enhance postoperative recovery and patient comfort.

## INTRODUCTION

Breast cancer remains the most frequently diagnosed malignancy among women worldwide and continues to represent a major public health concern. According to global cancer statistics, its incidence continues to rise despite improvements in screening and early detection strategies. Surgical management remains the cornerstone of treatment, and breast-conserving surgery (BCS) has become the standard approach for eligible patients with early-stage disease [1].

Recent meta-analyses have demonstrated comparable overall survival between mastectomy and BCS combined with adjuvant radiotherapy, reinforcing the oncological safety of conservative approaches. A comprehensive meta-analysis by Rajan *et al*. confirmed that BCS with adjuvant radiotherapy provides survival outcomes equivalent to mastectomy in early-stage breast cancer [2].

Despite its oncologic safety and cosmetic advantages, BCS is not free of postoperative complications. Hematoma, surgical site infection, wound dehiscence, persistent pain, and seroma formation are commonly observed. Among these, postoperative pain and seroma formation are particularly relevant due to their impact on quality of life, delay in adjuvant therapy, need for repeated interventions, and patient anxiety.

Seroma formation following breast and axillary surgery has been reported with highly variable incidence rates ranging from 3% to 85%, depending on surgical technique and definition criteria, as reported by Boostrom *et al*. Persistent seroma may necessitate repeated aspirations and has been associated with infection risk and delayed adjuvant therapy initiation [3].

Postoperative breast support has traditionally been recommended to minimize dead space, reduce tissue movement, and potentially decrease inflammatory exudation. However, high-quality evidence evaluating the clinical impact of supportive bra use after BCS remains limited. Although commonly advised in clinical practice, the objective benefits of consistent postoperative bra use on pain control and seroma resolution have not been sufficiently investigated in a structured comparative design.

Therefore, this single-center retrospective study aimed to evaluate the effect of regular supportive bra use on postoperative pain intensity and long-term seroma outcomes following breast-conserving surgery.

## Material and METHODS

### Study design and patient selection

This single-center retrospective cohort study included female patients who underwent upfront breast-conserving surgery between January 2023 and January 2025. Institutional approval was obtained before data collection.

Patients who met the predefined eligibility criteria were included in the study. The inclusion criteria were histologically confirmed breast carcinoma, upfront breast-conserving surgery, consistent use of a supportive bra, regular postoperative follow-up, and the availability of documented postoperative pain scores and ultrasonographic evaluations. Patients were excluded if they had received neoadjuvant therapy, had male breast cancer, had undergone surgery for benign breast disease, had irregular follow-up, or reported inconsistent use of a supportive bra during the postoperative period.

A total of 214 patients were initially screened. After excluding 31 patients who received neoadjuvant therapy, 16 patients with irregular follow-up or inconsistent supportive bra use, 33 patients who underwent segmental mastectomy for benign breast tumors, and 6 patients for other reasons, 128 patients were included in the final analysis.

### Postoperative supportive bra protocol

All patients received standardized postoperative instructions upon discharge. Use of a supportive bra was recommended for at least 16 hours daily for 3 months.

To minimize heterogeneity, supportive bras were categorized into two standardized types: medical-grade postoperative bras and compression sports bras providing mild-to-moderate uniform compression. Although exact pressure levels (mmHg) were not measured, all bras were selected based on their ability to provide consistent breast support without excessive constriction. Patients were instructed to avoid underwire bras and to use only full-coverage supportive models. Despite this effort toward standardization, variation in material and compression level remains a limitation of the study. Patients who did not comply with the defined usage protocol were excluded from the “user” group.

### Outcome measures

Pain intensity was assessed using the Visual Analog Scale (VAS) at postoperative week 1, month 1, month 6, and year 1. Patients’ postoperative pain was evaluated using a 10-point VAS, where 1 indicated no pain, and 10 represented the worst imaginable pain.

Seroma presence was evaluated clinically and confirmed by ultrasonography at 1-year follow-up, and was categorized as resolved (absent), reduced, or stable.

### Statistical analysis

All statistical analyses were conducted using IBM SPSS Statistics version 27 (IBM Corp., Armonk, NY, USA). Normality was assessed using the Kolmogorov–Smirnov test, which indicated non-normality of the variables.

Descriptive statistics were expressed as minimum, maximum, mean, standard deviation, median, and frequency where appropriate. Intergroup comparisons of quantitative variables were performed using the Kruskal–Wallis test, followed by Dunn’s post hoc analysis. The Mann–Whitney U test was used for two-group comparisons. Categorical variables were analyzed using the Fisher–Freeman–Halton exact test. Correlations were evaluated using Spearman’s ρ. A two-tailed *P* value <0.05 was considered statistically significant.

## RESULTS

### Patient characteristics

A total of 128 women who underwent BCS were included. The mean age was 54.64 ± 9.21 years (range: 36–72). Body mass index (BMI) ranged between 19.6 and 32.8 kg/m^2^ (mean: 27.09 ± 2.53 kg/m^2^).

As shown in Table 1, 56.3% of the participants had completed primary school, 23.4% middle school, 9.4% high school, and 10.9% university education. Comorbidities were present in 39.8% of the participants, while 10.9% were smokers. Most participants (96.1%) were right-hand dominant. Breast cancer was located on the left side in 59.4% of participants and on the right side in 40.6%. Regarding the relationship between hand dominance and cancer laterality, 44.6% of participants had ipsilateral involvement, whereas 55.4% had contralateral involvement (Table 1).

**Table 1:** Demographic and clinical characteristics of the participants (*n* = 128)

Variable	Category	*n*	%
Education level	Primary school	72	56.3
	Middle school	30	23.4
	High school	12	9.4
	University	14	10.9
Comorbidity	Yes	51	39.8
	No	77	60.2
Smoking status	Yes	14	10.9
	No	114	89.1
Dominant hand	Right	123	96.1
	Left	5	3.9
Breast cancer side	Left	76	59.4
	Right	52	40.6
Dominant hand and cancer laterality	Ipsilateral	57	44.6
	Contralateral	71	55.4

The pathological subtypes of the tumors were distributed as follows: 73.4% were Luminal A, 19.5% Luminal B, 4.7% HER2-positive, and 2.3% triple-negative (Table 2).

**Table 2:** Pathological subtype distribution

Subtype	*n*	%
Luminal A	94	73.4
Luminal B	25	19.5
HER2-positive	6	4.7
Triple-negative	3	2.3

Standard breast-conserving surgery (BCS) was performed in 71.9% of cases, while oncoplastic BCS (including techniques such as racket and batwing mastopexy) was performed in 28.1% of cases. A breast drain was used in 19.5% of patients. Sentinel lymph node biopsy (SLNB) was performed in 87.5% of cases, and axillary dissection in 12.5%; all patients who underwent axillary dissection had an axillary drain placed. All patients received postoperative radiotherapy. The mean specimen size was 6.24 ± 1.26 cm, with a median of 6.5 cm (range: 3–9 cm). Postoperatively, 57.8% of patients used a supportive bra, while 42.2% did not (Table 3).

**Table 3:** Surgical and postoperative characteristics

Variable	Category	*n*	%
BCS	Standard BCS	92	71.9
	Oncoplastic BCS	36	28.1
Drain in the breast site	Yes	25	19.5
	No	103	80.5
Axillary surgery	SLNB	112	87.5
	Axillary Dissection	16	12.5
Postoperative radiotherapy	Yes	128	100
Supportive bra use	Yes	74	57.8
	No	54	42.2
Specimen size (cm)		min-max	Mean ± SD (Median)
		3–9	6.24 ± 1.26 (6.5)

SLNB, Sentinel lymph node biopsy

**Figure 1:**
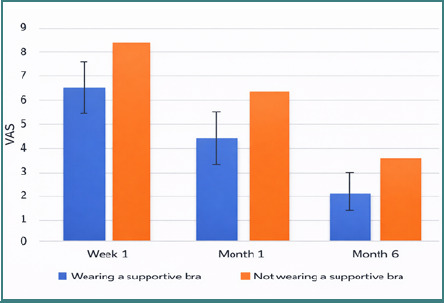
VAS scores according to supportive bra use

Patients who used a supportive bra had significantly lower VAS scores at postoperative week 1, month 1, and month 6 (all *P* = 0.001). No statistically significant difference was observed at year 1 (*P* = 0.052) (Table 4, Figure 1).

**Table 4:** VAS Scores according to supportive bra use

Time	Yes (*n* = 74) Mean ± SD (Median)	No (*n* = 54) Mean ± SD (Median)	*P*
1^st^ Week	6.38 ± 1.02 (6)	8.15 ± 1.02 (8)	0.001*
1^st^ Month	4.08 ± 1.13 (4)	6.15 ± 1.34 (6)	0.001*
6^th^ Month	2.07 ± 0.93 (2)	3.33 ± 1.05 (3)	0.001*
1^st^ Year	0.62 ± 0.68 (1)	0.78 ± 0.70 (1)	0.052

Mann–Whitney U Test; *P < 0.05

**Figure 2:**
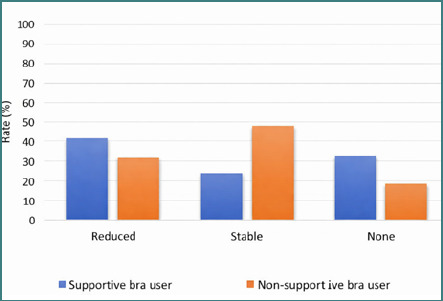
One-year seroma status according to supportive bra use

There was a statistically significant association between supportive bra use and the presence of seroma findings at the 1-year follow-up (*P* = 0.025; *P* < 0.05). The rate of absence of seroma at 1 year was significantly higher in patients who used a supportive bra (32.4%) than in those who did not (18.5%; Table 5, Figure 2).

**Table 5:** One-year seroma status according to supportive bra use

Seroma status	Bra *n* (%)	No bra *n* (%)	*P*
Stable	19 (25.6%)	27 (50.0%)	
Reduced	31 (41.9%)	17 (31.5%)	0.025*
Absent	24 (32.4%)	10 (18.5%)	

Education level was included as a demographic variable to explore its potential association with postoperative outcomes and patient compliance. There was no statistically significant difference in VAS scores at the 1^st^ week and 1^st^ year across educational levels (*P* > 0.05).

However, a statistically significant difference was observed in VAS scores at the 1^st^ month among different educational levels (*P* = 0.010; *P* < 0.05). Post hoc analysis using Dunn’s test was performed to determine the source of this difference. The 1^st^-month VAS scores of primary school graduates were significantly higher than those of high school graduates (*P* = 0.010; *P* < 0.05). No statistically significant differences were observed among the other educational groups in 1^st^-month VAS scores (*P* > 0.05).

A statistically significant difference was also found in VAS scores at the 6^th^ month across educational levels (*P* = 0.047; *P* < 0.05). The 6^th^-month VAS scores of primary school graduates were significantly higher than those of high school graduates (*P* = 0.048; *P* < 0.05). No significant differences were identified among the other educational groups regarding 6^th^-month VAS scores (*P* > 0.05; Table 6).

**Table 6:** Comparison of VAS scores according to education level

VAS	Primary school Mean ± SD (Median)	Middle school Mean ± SD (Median)	High school Mean ± SD (Median)	University Mean ± SD (Median)	*P*
Week 1	7.35 ± 1.32 (8)	7.03 ± 1.25 (7)	6.42 ± 1.00 (6)	6.79 ± 1.67 (6)	0.097
Month 1	5.26 ± 1.60 (5)	4.87 ± 1.46 (4.5)	3.75 ± 0.87 (4)	4.57 ± 1.83 (4)	0.010*
Month 6	2.79 ± 1.19 (3)	2.57 ± 1.10 (3)	1.83 ± 0.83 (2)	2.36 ± 1.15 (2)	0.047*
Year 1	1.04 ± 0.85 (1)	0.83 ± 0.79 (1)	0.58 ± 0.67 (0.5)	0.64 ± 1.01 (0)	0.092

Kruskal–Wallis Test; *P < 0.05

There was no statistically significant difference in VAS scores at the 1^st^ week, 1^st^ month, 6^th^ month, or 1^st^ year between patients with ipsilateral dominant hand involvement and those with contralateral dominant hand involvement (*P* > 0.05; Table 7).

**Table 7:** Comparison of VAS scores according to tumor laterality relative to hand dominance

VAS	Same side (*n* = 57) Mean ± SD (Median)	Different side (*n* = 71) Mean ± SD (Median)	*P*
Week 1	7.2 ± 1.38 (7)	6.98 ± 1.26 (7)	0.361
Month 1	5.01 ± 1.64 (5)	4.84 ± 1.49 (5)	0.543
Month 6	2.69 ± 1.19 (3)	2.42 ± 1.10 (2)	0.270
Year 1	0.95 ± 0.84 (1)	0.81 ± 0.85 (1)	0.331

* Mann–Whitney U Test

A statistically significant difference in 1^st^-week VAS scores was observed according to molecular subtype (*P* = 0.004; *P* < 0.05). Patients with the HER2-positive molecular subtype had significantly lower 1^st^-week VAS scores than those with the Luminal A (*P* = 0.021) and Luminal B (*P* = 0.003) subtypes (*P* < 0.05). No statistically significant differences were identified among the other molecular subtypes in terms of 1^st^-week VAS scores (*P* > 0.05).

Similarly, 1^st^-month VAS scores differed significantly across molecular subtypes (*P* = 0.010; *P* < 0.05). Patients with the HER2-positive subtype demonstrated significantly lower 1^st^-month VAS scores than those with Luminal A (*P* = 0.035) and Luminal B (*P* = 0.006) subtypes (*P* < 0.05). No other significant pairwise differences were observed between the remaining molecular subtypes (*P* > 0.05).

In contrast, no statistically significant differences were found in 6^th^-month or 1^st^-year VAS scores among the molecular subtypes (*P* > 0.05; Table 8).

**Table 8:** Comparison of VAS scores according to pathological subtypes

Time	Luminal A (*n* = 94) Mean ± SD (Median)	Luminal B (*n* = 25) Mean ± SD (Median)	HER2 positive (*n* = 6) Mean ± SD (Median)	Triple negative (*n* = 3) Mean ± SD (Median)	*P*
Week 1	7.13 ± 1.27 (7)	7.60 ± 1.47 (8)	5.50 ± 0.55 (5.5)	6.33 ± 0.58 (6)	0.004*
Month 1	4.94 ± 1.52 (5)	5.52 ± 1.76 (5)	3.17 ± 0.75 (3)	4.33 ± 0.58 (4)	0.010*
Month 6	2.59 ± 1.11 (3)	2.92 ± 1.35 (3)	2.00 ± 0.89 (2)	1.67 ± 0.58 (2)	0.152
Year 1	0.95 ± 0.87 (1)	0.92 ± 0.76 (1)	0.33 ± 0.52 (0)	0.67 ± 1.15 (0)	0.293

VAS, Visual Analog Scale; SD, Standard deviation. *P < .05.

VAS scores did not differ significantly across surgical approaches at the 1^st^ week, 1^st^ month, 6^th^ month, or 1^st^ year (*P* > 0.05; Table 9).

**Table 9:** Comparison of VAS scores according to type of breast-conserving surgery

Time	Standard BCS (*n* = 92) Mean ± SD (Median)	Oncoplastic BCS (*n* = 36) Mean ± SD (Median)	*P*
Week 1	7.07 ± 1.33 (7)	7.28 ± 1.37 (8)	0.439
Month 1	4.88 ± 1.60 (5)	5.14 ± 1.57 (5)	0.303
Month 6	2.67 ± 1.16 (3)	2.42 ± 1.16 (3)	0.345
Year 1	0.91 ± 0.85 (1)	0.89 ± 0.85 (1)	0.881

VAS, Visual Analog Scale; SD, Standard deviation.

Six-month VAS scores were significantly lower in patients with a breast surgical bed drain than in those without a drain (*P* = 0.043; *P* < 0.05).

In contrast, no statistically significant differences were observed between patients with and without a breast surgical bed drain in terms of VAS scores at 1 week, 1 month, or 1 year (*P* > 0.05; Table 10).

**Table 10:** Comparison of VAS scores according to drain use in the breast surgical cavity

Time	With drain (*n* = 25) Mean ± SD (Median)	Without drain (*n* = 103) Mean ± SD (Median)	*P*
Week 1	7.08 ± 1.41 (7)	7.14 ± 1.33 (7)	0.853
Month 1	4.92 ± 1.61 (5)	4.96 ± 1.60 (5)	0.924
Month 6	2.16 ± 1.07 (2)	2.71 ± 1.16 (3)	0.043*
Year 1	0.76 ± 0.66 (1)	0.94 ± 0.88 (1)	0.499

VAS, Visual Analog Scale; SD, Standard deviation. *P < .05.

VAS scores at 1 week, 1 month, 6 months, and 1 year did not differ significantly between patients who underwent sentinel lymph node biopsy (SLNB) and those who underwent axillary lymph node dissection (ALND) (*P* > 0.05; Table 11).

**Table 11:** VAS scores according to axillary surgery type over time

Time	SLNB (*n* = 112)	Axillary dissection (*n* = 16)	*P*
Week 1	7.11±1.34 (7)	7.25±1.39 (7.5)	0.722
Month 1	4.94±1.52 (5)	5.06±2.08 (5)	0.855
Month 6	2.60±1.15 (3)	2.63±1.26 (3)	0.896
Year 1	0.88±0.82 (1)	1.13±1.02 (1)	0.374

SLNB, Sentinel Lymph Node Biopsy; *Mann–Whitney U Test

No statistically significant correlation was identified between surgical specimen size and postoperative VAS scores at the 1^st^ week, 1^st^ month, 6^th^ month, or 1^st^ year (*P* > 0.05; Table 12).

**Table 12:** Relationship between specimen size and VAS scores over time

Time	Correlation (r)	*P*
1st week	0.089	0.319
1st month	0.079	0.378
6th month	-0.111	0.212
1st year	-0.135	0.128

At 1-year follow-up, a statistically significant difference in seroma status was observed according to education level (*P* = 0.009; *P* < 0.05). The rate of complete seroma resolution was higher among high school graduates (58.3%) and university graduates (42.9%) than among primary school graduates (22.2%) and middle school graduates (16.7%).

In contrast, no significant differences in 1-year seroma status were observed according to tumor laterality relative to the dominant hand, pathological subtype, type of breast-conserving surgery, postoperative drain placement in the breast surgical bed, or type of axillary surgical procedure (all *P* > 0.05; Table 13).

**Table 13:** Seroma status at 1-year follow-up by demographic and clinical variables

Variable	Decreased *n* (%)	Stable *n* (%)	Absent *n* (%)	*P*
**Education**				0.009*
Primary school	24 (33.3)	32 (44.4)	16 (22.2)	
Middle school	16 (53.3)	9 (30)	5 (16.7)	
High school	4 (33.3)	1 (8.3)	7 (58.3)	
University	4 (28.6)	4 (28.6)	6 (42.9)	
**Dominant hand vs. cancer side**				0.370
Ipsilateral	12 (22.2)	20 (37.0)	22 (40.7)	
Contralateral	39 (54.9)	21 (29.5)	11 (15.5)	
**Cancer pathology subgroup**				0.186
Luminal A	39 (41.5)	32 (31.9)	23 (24.5)	
Luminal B	7 (28)	12 (48)	6 (24)	
HER2 positive	0 (0)	2 (33.3)	4 (66.7)	
Triple negative	2 (66.7)	0 (0)	1 (33.3)	
**Surgical technique (BCS type)**				0.897
Standard BCS	35 (38.4)	33 (35.8)	24 (26.1)	
Oncoplastic BCS	13 (36.1)	13 (36.1)	10 (27.8)	
**Drain in the breast cavity**				0.840
Yes	8 (32)	9 (36)	8 (32)	
No	40 (38.8)	37 (35.9)	26 (25.2)	
**Axillary surgery**				0.292
SLNB	44 (39.3)	37 (31.3)	31 (27.7)	
Axillary dissection	4 (25)	9 (56.3)	3 (18.8)	

SLNB, Sentinel Lymph Node Biopsy; BCS, Breast Conserving Surgery.

## DISCUSSION

Breast-conserving surgery is currently performed safely in breast cancer surgery in accordance with established oncological principles [4]. As with all surgical procedures, complications such as hematoma, infection, seroma, wound dehiscence, and pain may occur after surgery [5]. These conditions can generally be controlled with close follow-up and appropriate treatment. However, prolonged postoperative complications may delay patients’ return to their daily routines.

Effective intraoperative hemostasis and adherence to sterile surgical techniques play an important role in preventing complications, particularly hematoma and infection. In addition to the use of analgesics, supportive bra use in the postoperative period has also been reported to have beneficial effects on postoperative pain in the literature. Consistent with these findings, our study demonstrated that regular bra use in the postoperative period had a statistically significant effect on postoperative pain [6-8].

Postoperative pain after breast cancer surgery is multifactorial and may be influenced by surgical technique, axillary intervention, inflammatory response, psychological factors, and pre-existing pain perception. Approximately 10% of patients develop chronic persistent pain following breast cancer surgery, as reported by Driul *et al*. Early postoperative pain intensity has been shown to correlate with long-term pain outcomes, emphasizing the importance of early pain control strategies [9].

This study demonstrates that regular use of supportive bras following BCS is associated with significantly lower postoperative pain during the early and intermediate postoperative periods (week 1, month 1, and month 6). Although pain scores converged by the first postoperative year, the early reduction in pain is clinically meaningful, as this period corresponds to the most functionally limiting stage of recovery.

Seroma formation remains one of the most frequent sequelae of breast surgery. Seroma formation is a common postoperative complication following breast cancer surgery and occurs in approximately 3% to 85% of patients. The reported incidence of seroma formation ranges from 29% to 36.5% in patients undergoing modified radical mastectomy (MRM). In contrast, it varies between 18% and 26.8% in those treated with breast-conserving surgery combined with axillary dissection (BCS + AD). As described by Boostrom SY *et al.*, clinically significant seroma can lead to patient discomfort and repeated interventions. Although a seroma is often considered a benign postoperative finding, its prolonged persistence may delay adjuvant treatment and increase the risk of infection [3,10].

The mechanical stabilization provided by supportive bras may reduce traction forces on healing tissues, limit excessive motion within the surgical cavity, and decrease inflammatory stimulation. Additionally, controlled compression may help reduce fluid accumulation in the postoperative dead space [11]. In our cohort, supportive bra users demonstrated a significantly higher rate of complete seroma resolution at one year compared to non-users. While short-term seroma development was not the primary endpoint, long-term absence of seroma suggests that sustained compression may facilitate gradual resorption of accumulated fluid.

Interestingly, specimen size, surgical type (standard vs. oncoplastic BCS), axillary surgery type, and pathological subtype were not significantly associated with long-term pain outcomes. These findings suggest that mechanical stabilization provided by supportive garments may play a more relevant role in early recovery than intrinsic tumor-related factors.

Educational level was associated with differences in intermediate VAS scores and long-term seroma resolution. Although education level was not a primary variable of interest, its association with postoperative outcomes may reflect differences in health literacy, treatment adherence, and compliance with postoperative instructions, such as regular use of a supportive bra. Patients with higher educational levels may better understand and adhere to postoperative care recommendations, which could indirectly influence clinical outcomes. Therefore, education level may act as a surrogate marker for patient compliance rather than a direct biological determinant.

The non-invasive nature, low cost, and ease of implementation of supportive bra use make it an attractive adjunct in postoperative care. Unlike pharmacologic pain control, it carries no systemic side effects and may enhance patient comfort without additional resource burden.

The possible mechanism underlying reduced seroma persistence in supportive bra users may involve controlled external compression, which decreases dead space volume and limits shear forces between dermoglandular flaps. Reduced mechanical motion may attenuate the production of inflammatory exudate and enhance lymphatic reabsorption [12-14].

To our knowledge, this is one of the first studies specifically evaluating the long-term impact of structured supportive bra use on both postoperative pain trajectory and seroma resolution following breast-conserving surgery.

### Strengths and limitations

This study has several strengths, including its single-center design and the use of standardized follow-up intervals for patient evaluation. Nevertheless, certain limitations should be considered when interpreting the findings. First, the retrospective design and the absence of randomization may have introduced selection bias. Additionally, adherence to bra use was based on patient self-reporting, which may have affected the reliability of the data. Another limitation is the lack of objective standardization regarding the material properties and compression levels of the supportive bras used by patients. Furthermore, quality-of-life outcomes were not evaluated. Future prospective, randomized studies are therefore required to validate these findings and to establish optimal compression parameters.

From a clinical standpoint, early pain reduction may facilitate faster mobilization, improved shoulder function, and better adherence to adjuvant radiotherapy schedules. Given that the first six postoperative months represent the most vulnerable recovery phase, interventions that reduce pain during this window may indirectly improve functional recovery and psychological well-being.

## CONCLUSION

Regular use of a supportive bra following breast-conserving surgery is associated with significantly reduced early postoperative pain and improved long-term seroma resolution. Given its simplicity, non-invasive nature, and minimal cost, supportive bra use should be considered as part of routine postoperative care after BCS.
